# Supplementary material for: Shoreface erosion counters blue carbon accumulation in transgressive barrier-island systems
Source: Nat Commun. 2023 Dec 19;14:8425. doi: 10.1038/s41467-023-42942-8 (PMC10730579; doi:10.1038/s41467-023-42942-8)
Supplement: Supplementary file 1 — Supplementary Information [file 41467_2023_42942_MOESM1_ESM.pdf]

Shoreface erosion counters blue carbon accumulation in transgressive barrier-island systems

Mary Bryan Barksdale[1,]*, Christopher J. Hein[1], Matthew L. Kirwan[1]

[1]*Virginia Institute of Marine Science, William & Mary, P.O. Box 1346, Gloucester Point, VA 23062 USA*

* - corresponding author (mbarksdale@vims.edu)

Supplementary Information

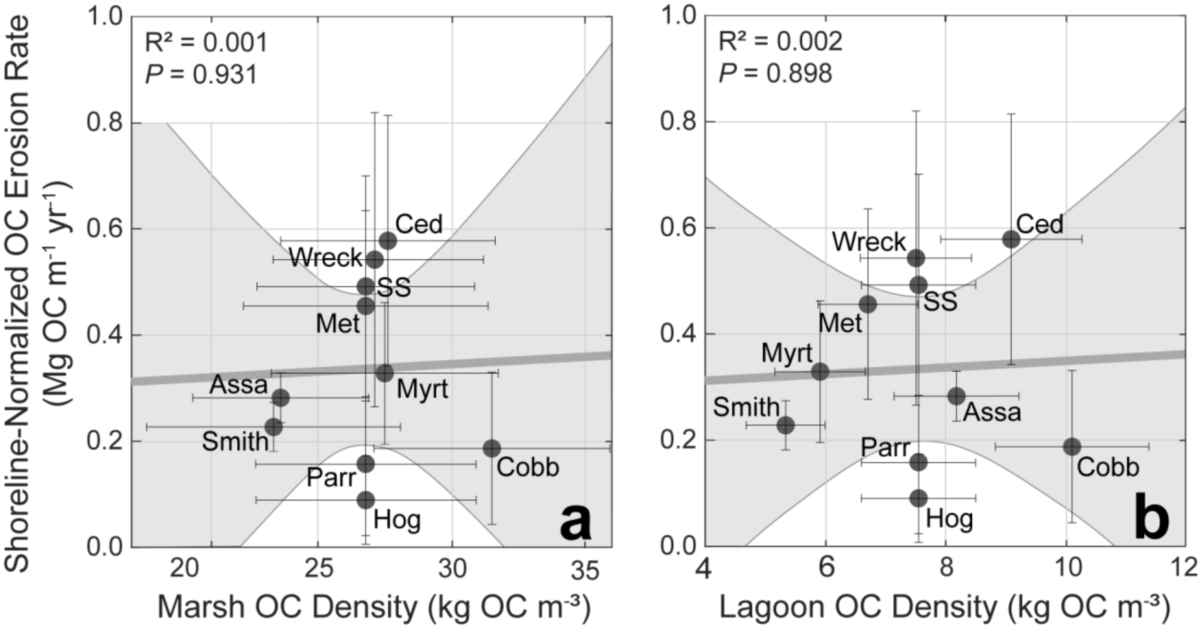

Supplementary Figure 1. Regressions between average (1870–2017 C.E.) shoreline-normalized organic carbon (OC) erosion rates and **a** Marsh OC density and **b** Lagoon OC density. Solid lines indicate fitted linear regressions; gray windows demarcate 95% confidence intervals. Island abbreviations: Assa=Assawoman; Met=Metompkin; Ced=Cedar; Parr=Parramore; SS=Ship Shoal; Myrt=Myrtle.

1. Total Organic Carbon (TOC) and Organic Matter (OM) Values and Uncertainties

We analyzed a subset of sediment subsamples for total organic carbon (TOC) on a Costech Elemental Analyzer, model 4010, coupled to a ThermoFisher DeltaV Isotope Ratio Mass Spectrometer to determine conversion factors for organic matter (OM) to organic carbon (OC) (Supplementary Fig. 2). Prior to analysis, we freeze-dried and powdered samples and removed

carbonates by adding 2 drops of 1N HCl and drying overnight at 60°C following methods outlined in refs. [1,2]. Average analytic precision (2-σ) for replicate measurements of marsh and lagoon TOC, respectively, were 0.06% and 0.18%. Uncertainties for all OC values were based on 95% confidence intervals and were propagated through to uncertainty estimates for final OC erosion rates.

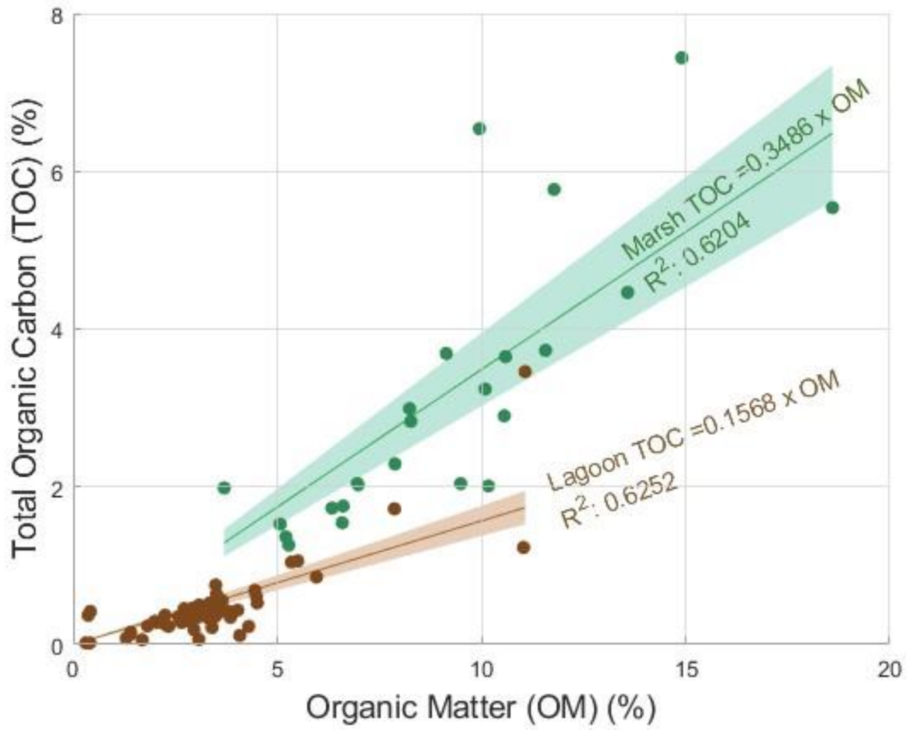

Supplementary Figure 2. Marsh- (green) and lagoon- (brown) specific organic matter (OM) and total organic carbon (TOC) values from sediment core subsamples. Linear regressions (solid lines) were forced through the origin. Green and brown shaded windows demarcate 95% confidence intervals.

2. Depth-Dependent Shoreface Erosion Rates

Morphologic change along the deep shoreface (that is, above the shoreface toe but below the zone of sediment transport by fair-weather waves) in response to sea-level rise (SLR) or to barrier-island transgression has not been well constrained on observational timescales[3,4]. Because sediment erosion and transport decrease following depth-dependent reductions in wave activity,

deeper reaches of the shoreface may respond minimally to, and/or temporally lag, changes occurring in the upper or mid-shoreface[4]. This timescale-dependence is partially due to the increase in depth of closure over longer timescales (multi-decadal to centuries), during which less-frequent but more-powerful storms interact with deeper reaches of the shoreface[3,5]. However, for the upper and middle shorefaces, morphologic change in response to dynamic sea level and barrier-island movement is expected to occur on decadal to centennial timescales[4].

Given an estimated regional depth of closure (that is, shoreface toe) of 10–15 m for the Virginia Barrier Islands (VBI)[3,6,7]—which is more than 1.5 m greater than the system-wide average depth of the Holocene lagoon (Supplementary Table 5)—we assume that all marsh and lagoon sediments are fully eroded on the beach and shoreface over the multi-decadal to centennial timescales of interest in this study. Accordingly, we assume a fully erosional transgression, in which all backbarrier deposits are eventually eroded along the shoreface; albeit deeper basal lagoonal sediments are not eroded until exposed at depth, some kilometers offshore. This assumption is supported by recent bathymetric and seismic mapping along the VBI, which demonstrate that—apart from thick inlet-fill deposits—Holocene sediments thin seaward across the shoreface and are fully eroded within 0.5 km of the modern beach, leaving only a discontinuous thin veneer of sandy lag atop Pleistocene deposits on the shallow shelf[8,9].

Finally, we note that our approximations of carbon erosion are conservative. Firstly, the thickness of shoreface-eroded Holocene sediments is likely a minimum estimate: for only few islands were we able to collect cores that penetrated to underlying Pleistocene deposits. For all others, we use the (likely incomplete) Holocene lagoon unit thickness that was captured; thus, the actual lagoon thicknesses for some islands may exceed those used in our analyses. Secondly, we note that only the carbon losses from Holocene deposits is considered in our analysis. For

example, refs. [7,10] report likely erosion and reworking of sandy Pleistocene deposits along the VBI shoreface, re-exposing any OC within these sediments to remineralization and/or export; these are unaccounted for in our budgets, but any erosion of these deeper, relatively carbon-poor deposits would add to shoreface carbon erosion.

3. Calculation of Shoreline-Change Rates, Shoreline Lengths, and Uncertainties

Shoreline-change rates are based on outputs from the Digital Shoreline Analysis System[11] (DSAS). Island-averaged shoreline change rates are determined from either linear regression rates or end-point-rates of shoreline change for all transects for a given island. For shorelines mapped before 1870, we interpolated shoreline position at year 1870 by using the end-point-rate between survey years directly before and directly after 1870. We used these interpolations as the new starting shoreline position for any time periods beginning with 1870. For time periods considered within this study (that is, 1870–2017, 1870–1942, 1942–1994, 1994–2017), we used the average linear regression rate of island transects only when more than four shoreline positions were available for at least 75% of all island transects; otherwise, we took the average end-point rate between earliest and most recent shoreline-position datapoint for all transects along an island. Each method has drawbacks and advantages[12]. Specifically to our work, the maximum end-point-rate in a time period may be the most accurate way to account for total (non-annualized) lagoon OC erosion, as we assume all transgression results in erosion of lagoon sediments that cannot be recovered during periods of island widening. However, the linear regression rate approach to estimating shoreline change is likely the most accurate method because it accounts for four or more shoreline positions within a given time interval. Therefore, we use linear regression rates when possible, recognizing that the result is a conservative

approximation of shoreline change rates and therefore a conservative estimate of annual lagoon OC erosion. Refer to footnotes of Supplementary Table 2 for more information about uncertainty calculations.

Because the lagoon OC erosion term (equation 1 in manuscript; Table 1 in manuscript) depends in part on the shoreline change rate, for time periods during which an island experiences an average negative shoreline-change rates (net growth), the lagoon OC erosion term becomes 0 Gg OC yr^{-1}. Additionally, we subtracted the equivalent lagoon volume covered during a period characterized by net shoreline regression (island widening) from the volume exposed during the next transgressive period, as a conservative accounting of lagoon previously eroded. Compared to our methodology for approximating lagoon OC erosion, the greater spatial resolution and poorer temporal resolution of our approach for assessing island-wide marsh exposure rates (for example, accounting for sub-island-scale marsh exposure along the beach and shoreface; see Section 4 below for more detail) means that islands with periods of negative average shoreline change rates (that is, net regression), may still contribute, albeit minimally, to the VBI OC erosion term.

Supplementary Table 1. Shoreline Lengths ($L_{shoreline}$)

Island	$L_{shoreline1870}$ (m)	$L_{shoreline1942}$ (m)	$L_{shoreline1994}$ (m)	$L_{shoreline2017}$ (m)
Assawoman	6599 ± 16	6935 ± 16	5260 ± 8	5120 ± 4
Metompkin	11442 ± 16	10204 ± 16	11096 ± 8	10075 ± 4
Cedar	10687 ± 16	9781 ± 16	12093 ± 8	11495 ± 4
Parramore	13000 ± 16	13457 ± 16	12372 ± 8	12070 ± 4
Hog	11288 ± 16	11041 ± 16	12877 ± 8	12478 ± 4
Cobb	10256 ± 16	9131 ± 16	8847 ± 8	6679 ± 4
Wreck	3934 ± 16	5741 ± 16	7106 ± 8	5278 ± 4
Ship Shoal	3405 ± 16	4407 ± 16	4187 ± 8	4319 ± 4
Myrtle	3659 ± 16	4310 ± 16	3778 ± 8	3321 ± 4
Smith	12633 ± 16	12067 ± 16	11373 ± 8	11631 ± 4

Note: Shoreline lengths are from ref. [13]. Shorelines terminate at the point where they shift ~90° away from the predominant open-ocean orientation. Uncertainties are those introduced during mapping of both shoreline terminuses of a given island, following ref. [12], with shorelines derived from T-sheets (that is, surveys conducted in 1870 and 1942) introducing 11.7 m uncertainty, those from air photos (that is, surveys conducted in 1994) introducing 5.5 m uncertainty, and those derived from lidar (that is, 2017) introducing 2.3 m uncertainty.

Supplementary Table 2. Shoreline Change Rates (*SCR*)

Island	1870–2017 LRR* ($m\ yr^{-1}$)	1870–1942* EPR† ($m\ yr^{-1}$)	1942–1994 EPR† ($m\ yr^{-1}$)	1994–2017 LRR* ($m\ yr^{-1}$)
Assawoman	4.74 ± 0.68	2.23 ± 0.25	6.74 ± 0.23	4.56 ± 3.66
Metompkin	7.67 ± 1.45	4.57 ± 0.24	11.23 ± 0.22	3.54 ± 3.80
Cedar	6.68 ± 1.37	4.04 ± 0.24	5.47 ± 0.23	15.75 ± 4.78
Parramore	5.94 ± 1.82	3.19 ± 0.27	8.86 ± 0.22	11.93 ± 3.75
Hog	3.10 ± 1.95	8.10 ± 0.25	-1.30 ± 0.23	1.12 ± 3.94
Cobb	3.03 ± 2.77	3.61 ± 0.29	2.48 ± 0.22	13.63 ± 7.69
Wreck	5.72 ± 3.21	9.14 ± 0.39	5.78 ± 0.22	-5.35 ± 13.42
Ship Shoal	8.07 ± 3.14	0.67 ± 0.25	16.67 ± 0.22	-3.23 ± 9.61
Myrtle	6.38 ± 2.35	5.64 ± 0.24	3.03 ± 0.22	30.99 ± 6.26
Smith	5.63 ± 1.01	6.81 ± 0.24	3.59 ± 0.23	8.04 ± 4.23
All Islands	5.57 ± 1.79	5.04 ± 0.26	5.82 ± 0.22	7.88 ± 5.17

Note: Negative values indicate regression (that is, seaward translation) of the oceanside shoreline (beach growth), while positive values indicate transgression (erosion and/or island migration).

*Following ref. [12], uncertainties calculated as the average of all transect 90% confidence intervals between oldest and youngest shorelines in a given range.

†Uncertainties calculated as the average of all transect EPR uncertainties, which, following ref. [12] are equal to the quadrature addition of mapping uncertainties associated with both shoreline positions (that is, oldest and youngest positions within the time range) divided by the difference in years between shoreline surveys.

4. Marsh-Exposure Rate Methods and Uncertainties

Marsh-exposure rates, ER_{marsh}, for specific time periods (Supplementary Table 3) were calculated in three steps. First, using ArcGIS shapefiles from refs. [14,15], we calculated the areal extent of historical marsh exposed on the oceanside of the island at each endpoint time (that is, by 1942, 1994, and 2017). For each time period, the 1870 extent of backbarrier marsh constituted the historical marsh used as the baseline, following the methods of ref. [14]. Because islands in their earliest-mapped positions (surveyed between 1851 and 1888) likely were situated at that time atop some (unmeasurable) area of backbarrier marsh (marsh which would later have been exposed and eroded during subsequent transgression), the second step in estimating ER_{marsh} involved calculating the percent of the 1942 area of each island that overlaid marsh mapped in the original T-sheets. This percentage was then applied to the island area at the earliest mapped

year. For example, if 45% of a given island area in 1942 overlapped with marsh mapped in earlier years, we assumed that 45% of that island was underlain by marsh; we simply applied that same percentage to the original 1800s island area (that is, assumed that 45% of the island area was underlain by marsh at the time of earliest mapping). We then assume that this buried marsh from the 1800s was exposed and eroded in accordance with barrier migration over time. Lastly, to estimate marsh-exposure rates, we interpolated the total area of shoreface-exposed marsh over certain time periods (that is, 1870–2017, 1870–1942, 1942–1994, 1994–2017) to an annual rate. Because islands were first mapped over a course of 37 years (1851–1888), we used the annual marsh exposure rate to interpolate total areal loss between a single, normalized year (1870) and each subsequent year of interest (that is, 1942, 1994, and 2017). Comparing total losses between 1870 and each subsequent year allowed us to calculate total marsh exposure and annual marsh exposure rates for each time period (reported in the first column under each time interval in Supplementary Table 3).

It is possible portions of marsh mapped in the 1800s may have been eroded in the backbarrier before being buried by the adjacent landward-migrating islands: though still contributing to marsh and blue carbon loss, this component would be unrelated to barrier migration. We therefore include in our uncertainty of marsh-exposure rates a maximum potential amount of backbarrier marsh erosion, based on historic VBI marsh area and marsh loss rates reported in ref. [14], which computes to an annual VBI marsh erosion rate of 0.12%. Backbarrier marsh erosion is expressed on a per-island basis in Supplementary Table 3 under the headers 'Uncertainty of extent of backbarrier erosion.' Uncertainties associated with mapping are reported in Supplementary Table 3 under the column headers 'Uncertainty of exposed marsh

area' and are based on the 4.5% uncertainty associated with mapping the historic marsh area reported in ref. [14] (see Supplementary Table 3 footnotes for more details).

Supplementary Table 3. Marsh Exposure Rates (ER_{marsh}) and Associated Uncertainties

Island	1870–2017				1870–1942			
	Area of historic (1870) marsh exposed seaward of island by 2017 (m^2)	Uncertainty of exposed marsh area* (m^2)	Uncertainty of extent of backbarrier erosion† ($m^2\ yr^{-1}$)	Total uncertainty associated with ER_{marsh} ($m^2\ yr^{-1}$)	Area of historic (1870) marsh exposed seaward of island by 1942 (m^2)	Uncertainty of exposed marsh area* (m^2)	Uncertainty of extent of backbarrier erosion† ($m^2\ yr^{-1}$)	Total uncertainty associated with ER_{marsh} ($m^2\ yr^{-1}$)
Assa	2093775	96314	17	672	933113	42923	16	612
Met	7810864	359300	64	2508	3825729	175984	64	2508
Cedar	6134952	282208	50	1970	2673999	123004	45	1753
Parra	23679	1089	0	8	3415	157	0	2
Hog	554963	25528	5	178	269226	12384	4	176
Cobb	2338612	107576	19	751	981369	45143	16	643
Wreck	3234579	148791	26	1039	1584284	72877	26	1039
SS	1827466	84063	15	587	820982	37765	14	538
Myrtle	2240233	103051	18	719	1096091	50420	18	719
Smith	6833145	314325	56	2194	3326033	152998	55	2180

Note: Island abbreviations are: Assa = Assawoman; Met = Metompkin; Parra = Parramore.

*Assuming average uncertainty reported in ref. [14] of ± 4.5% (± 15.1km^2) for 1870 barriershed marsh area uncertainties, which was calculated by multiplying the perimeter of mapped marsh by the uncertainty introduced during mapping following ref. [12].

† Assuming minimum 1870 marsh extent and maximum interior marsh loss (lower and upper limits of uncertainties) reported by ref. [14] to encapsulate largest possible percent marsh loss from erosion preceding barrier island burial

Supplementary Table 3 Continued.

Island	1942–1994				1994–2017			
	Area of historic (1870) marsh exposed seaward of island between 1942 and 1994	Uncertainty of exposed marsh area*	Uncertainty of extent of backbarrier erosion[ƚ]	Total uncertainty associated with ER_{marsh}	Area of historic (1870) marsh exposed seaward of island between 1994 and 2017	Uncertainty of exposed marsh area*	Uncertainty of extent of backbarrier erosion[ƚ]	Total uncertainty associated with ER_{marsh}
	(m^2)	(m^2)	($m^2\ yr^{-1}$)	($m^2\ yr^{-1}$)	(m^2)	(m^2)	($m^2\ yr^{-1}$)	($m^2\ yr^{-1}$)
Assa	344650	15854	8	313	816012	37537	43	1675
Met	2215872	101930	51	2011	1769263	81386	92	3631
Cedar	633656	29148	15	575	2827297	130056	148	5802
Parra	10587	487	0	10	9677	445	1	20
Hog	239883	11035	6	218	45854	2109	2	94
Cobb	323774	14894	7	294	1033470	47540	54	2121
Wreck	1536853	70695	35	1395	113443	5218	6	233
SS	998639	45937	23	906	7845	361	0	16
Myrtle	0* (-185209)	0* (8520)	0*	0*	1144142[ƚ]	53316[ƚ]	60	2378
Smith	1568718	72161	36	1424	1938394	89166	101	3978

Note: Refer to notes in table above.

*A greater area of marsh became exposed seaward of Myrtle from 1870 to 1942 than from 1870 to 1994, leading to a calculation of 0 m^2 marsh loss from 1942 to 1994.

[ƚ] The difference between total marsh exposed between 1870–1942 and 1870–1994 (that is, 185,209 m^2) was subtracted from the area exposed between 1994–2017, as a conservative accounting of marsh eroded from 1870–1942 and therefore not available for erosion from 1994–2017.

5. Carbon Accumulation Rate Methods and Uncertainties

We use land-use extent and carbon accumulation rate (CAR) data from refs. [16–18] to calculate average CAR values (1984–2020 C.E.) for the VBI backbarrier. We adapted mapping data from ref. [21] to our more limited study area (northern Assawoman to southern Smith islands) and computed uncertainties based on propagations of standard error that were reported in the ref. [21] supplementary information. Unlike ref. [16], we considered VBI backbarrier lagoons as sites of OC accumulation. This reflects our new finding of the substantial contribution of lagoon sediment OC to total shoreface OC erosion. To estimate CAR for lagoons, we relied on OC content of VBI lagoon sediments reported by ref. [17] and sedimentation rates of a VBI lagoon reported by ref. [18]. Considering the lack of spatial data on lagoon CAR coupled with the lack of standard error reporting by refs. [18,19], uncertainties associated with our lagoon CAR are likely underestimates.

Supplementary Table 4. Estimated Annual Carbon Accumulation Rates (CAR) for the Virginia Backbarrier Marshes, Seagrass Beds, and Lagoons.

Habitat	VBI Backbarrier Area* (km^2)	CAR† (g OC m^{-2} yr^{-1})	Total habitat CAR (Gg OC yr^{-1})	Average CAR between 1984 and 2020 (Gg OC yr^{-1})
Marsh	1984: 273.5 ± 14.2<br>2020: 264.7 ± 9.7	78.4 ± 18.9*	1984: 21.4 ± 5.3<br>2020: 20.8 ± 5.1	21.1 ± 5.2
Seagrass	1984: 0.0<br>2020: 29.3	40.1 ± 2.9*	1984: 0<br>2020: 1.2 ± 0.3	0.6 ± 0.2
Lagoon	1984: 473.8 ± 31.0<br>2020: 445.4 ± 20.0	26.4§	1984: 12.5 ± 3.2<br>2020: 11.7 ± 2.5	12.1 ± 2.9
Total			1984: 33.9 ± 6.2<br>2020: 33.7 ± 5.7	33.8 ± 6.0

Note: These data feed into Fig. 3 and are used as a comparison to the average annual OC erosion rate of the VBI from 1994–2017 C.E.

*Values adapted from ref. [16] to extend from Assawoman in the north to Smith in the south, excluding all other VBI.

† Average CAR values include above- and belowground biomass and soil OC. CAR values from ref. [16] and uncertainties adapted from ref. [16].

§Combined average VBI lagoon %TOC from ref. [17] and average VBI lagoon sedimentation rates from ref. [18]; no standard error reported.

6. Sediment Core Data and Uncertainties

All cores collected as part of this study were opened, described, photographed, and sampled at a 10-cm resolution (for depths less than approximately 55 cm) in marsh peat and at 20–25 cm resolution through underlying lagoon deposits. We analyzed sample aliquots for organic-matter content (loss-on-ignition; LOI) and continued with the methods outlined in the main text.

We extended our analysis of carbon-loss rates to islands for which we were unable to collect new sediment cores through incorporation of island stratigraphy available in the literature (Supplementary Table 5). Values for 'Average of migrating islands' (Supplementary Table 5) were used to inform the OC erosion rate calculation for Ship Shoal (eq. (1) in manuscript). Likewise, Parramore averages were used to inform OC erosion rate values for Hog. Values for which Parramore Island data were not available (for example, OC densities and Holocene marsh thickness) were based on values calculated for ‘Average of migrating islands.’ Values for 'Average of migrating islands' exclude Parramore and Hog, which largely exhibit narrowing rather than migrating behavior[15].

With the exception of Met_G which reveals an anomalously thick Holocene unit (interpreted as a relict tidal inlet), cores collected from Assawoman, Metompkin, and Smith Islands as part of this study and others did not penetrate to the Pleistocene. Thus, we use all cores to inform stratigraphy and consider our lagoon thickness estimates for these islands to be conservative. This is in direct contrast to Cedar, where although cores collected as part of this study did not penetrate to the Pleistocene, we can approximate lagoon thickness from the two cores from the literature that did.

Sandy facies interbedded within the lagoon unit—likely relict washover fans or tidal inlets—

were considered a part of the “lagoon” deposits and thus integrated into the lagoon thickness and average OC density values, increasing the former but decreasing the latter (Fig. 1C).

Supplementary Table 5. Core Dataset.

Island	Core ID	Source	Latitude	Longitude	BACKBARRIER MARSH Thickness (m)	Average Thickness (When Present) (m)	OC Density (kg OC m^{-3})	Average OC Density (kg OC m^{-3})
Assa	Assa_1	this study	37.78816	-75.52742	1.01	1.26 ± 0.25	23.6 ± 3.3	23.6 ± 3.3
	1-1	Finkelstein (1986)[19]	37.81*	-75.52*	1.50		-	
Met	Met_1	this study	37.76783	-75.54031	0.82	0.66 ± 0.16	26.8 ± 3.9	26.8 ± 3.9
	Met_G	this study	37.71878	-75.57162	0.00		-	
	2-1	Finkelstein & Ferland (1987)[20]	37.75	-75.56	0.50		-	
Cedar	Ced_1	this study	37.66452	-75.59285	0.80	1.07 ± 0.27	25.9 ± 3.7	27.6 ± 4.0
	Ced_2	this study	37.62816	-75.60686	1.33		29.3 ± 4.3	
	CEDG01	Shawler et al. (2019)[21]	37.65545	-75.59554	0.00		-	
	CEDG03	Shawler et al. (2019)[21]	37.59447	-75.61875	0.00		-	
Parra	PARG03	Raff et al. (2018)[22]	37.55209	-75.61749	0.00	-	-	-
	PARG07	Raff et al. (2018)[22]	37.52491	-75.64808	0.00		-	
Cobb	Cobb_1	this study	37.30289	-75.77211	0.98	0.98	31.5 ± 4.4	31.5 ± 4.4
Wreck	WI_1	this study	37.28251	-75.79819	2.06	1.18 ± 0.88	27.1 ± 3.9	27.1 ± 3.9
	SDH-17-72	Halsey (1978)[23]	37.28528	-75.78581	0.30		-	
Myrtle	Myrt_2_02	this study	37.18726	-75.82460	0.60	0.60	27.5 ± 4.3	27.5 ± 4.3
Smith	Smith_1	this study	37.16908	-75.84273	0.50	0.56 ± 0.24	27.2 ± 6.4	23.3 ± 4.8
	Smith_2	this study	37.13960	-75.87531	0.17		19.4 ± 3.1	
	6-1	Finkelstein (1986)[19]	37.15*	-75.86*	1.00		-	
Average of all islands						0.79 ± 0.36		
Average of migrating islands						0.90 ± 0.36		26.8 ± 4.1

Note: Island abbreviations are: Assa=Assawoman; Met.=Metompkin; Parra.=Parramore; S.S.=Ship Shoal. Where data are absent for a given unit, that unit was either non-existent in a given core (for example, no marsh or sand), values were not given in original sources (for example, carbon data from published cores), or certain core values were not used to compute island averages (for example, Ced_1 and Ced_2 lagoon unit thicknesses were excluded, for reasons explained Section 6 above).

* Core locations were not reported in original publication and are based here on best available information.

Supplementary Table 5 Continued

Island	Core ID	SAND* Thickness (m)	SAND* Average Thickness (m)	LAGOON Thickness (m)	LAGOON Average Thickness (m)	LAGOON Base Depth (From Top of Core) (m)	LAGOON Average Base Depth (From Top of Core) (m)	LAGOON OC Density (kg OC m^{-3})	LAGOON Average OC Density (kg OC m^{-3})	Penetrate to Base of Holocene?
Assa	Assa_1	0.00	0.0 ± 0.0	6.01	5.63 ± 0.38	7.02	6.89 ± 0.19	8.2 ± 1.0	8.2 ± 1.0	N
	1-1	0.00		5.25		6.75		-		N
Met	Met_1	3.63	1.63 ± 1.06	4.16	7.26 ± 3.06	4.98	9.33 ± 4.46	4.1 ± 0.5	6.7 ± 0.8	N
	Met_G	0.00		13.37		18.25		9.3 ± 1.2		Y
	2-1	1.25		4.25		4.75		-		N
Cedar	Ced_1	0.57	1.64 ± 1.46	5.11	-	-	-	8.9 ± 1.2	9.1 ± 1.2	N
	Ced_2	0.00		1.97	-	-	-	9.3 ± 1.2		N
	CEDG01	0.00		4.25	7.63 ± 3.38	4.25	7.50 ± 3.25	-		Y
	CEDG03	6.00		11.00		10.75		-		Y
Parra	PARG03	1.00	0.50 ± 0.50	6.25	3.50 ± 2.75	11.00	10.88 ± 0.13	-	-	Y
	PARG07	0.00		0.75		10.75		-	-	Y
Cobb	Cobb_1	0.00	0.00	4.55	4.55	5.53	5.53	10.1 ± 1.3	10.1 ± 1.3	Y
Wreck	WI_1	0.00	1.38 ± 1.38	5.65	8.47 ± 2.82	7.71	9.65 ± 1.94	7.5 ± 0.9	7.5 ± 0.9	Y
	SDH-17-72	2.75		11.28		11.58		-		Y
Myrtle	Myrt_2_02	0.00	0.00	6.90	6.90	7.50	7.50	5.9 ± 0.8	5.9 ± 0.8	Y
Smith	Smith_1	2.25	1.11 ± 0.65	6.48	5.98 ± 0.29	6.98	6.79 ± 0.62	6.3 ± 0.8	5.3 ± 0.7	N
	Smith_2	1.08		5.46		5.63		4.4 ± 0.5		N
	6-1	0.00		6.00		7.75		-		N
Average of all islands			0.78 ± 0.84		6.04 ± 2.11		8.01 + 1.76			
Average of migrating islands			0.82 ± 0.91		6.63 ± 1.98		7.60 + 2.09		7.6 ± 1.0	

Note: Refer to notes in table above.

Supplementary References:

1. Verardo, D. J., Froelich, P. N. & McIntyre, A. Determination of organic carbon and nitrogen in marine sediments using the Carlo Erba NA-1500 analyzer. *Deep Sea Res. Part A Oceanogr. Res. Pap.* **37**, 157–165 (1990).
2. Nieuwenhuize, J., Maas, Y. E. M. & Middelburg, J. J. Rapid analysis of organic carbon and nitrogen in particulate materials. *Mar. Chem.* **45**, 217–224 (1994).
3. Ortiz, A. C. & Ashton, A. D. Exploring shoreface dynamics and a mechanistic explanation for a morphodynamic depth of closure. *J Geophys Res Earth Surf* **121**, 442–464 (2016).
4. Cowell, P. J. & Kinsela, M. A. Shoreface controls on barrier evolution and shoreline change. in *Barrier Dynamics and Response to Changing Climate* (eds. Moore, L. J. & Murray, A. B.) 243–275 (2018). doi:10.1007/978-3-319-68086-6_8.
5. Nicholls, R. J., Birkemeier, W. A. & Lee, G. Evaluation of depth of closure using data from Duck, NC, USA. *Mar. Geol.* **148**, 179–201 (1998).
6. Lorenzo-Trueba, J. & Mariotti, G. Chasing boundaries and cascade effects in a coupled barrier-marsh-lagoon system. *Geomorphology* **290**, 153–163 (2017).
7. Shawler, J. L. *et al.* Relative influence of antecedent topography and sea-level rise on barrier-island migration. *Sedimentology* **68**, 639–669 (2021).
8. Brothers, L. L., Foster, D. S., Pendleton, E. A. & Baldwin, W. E. Seismic stratigraphic framework of the continental shelf offshore Delmarva, U.S.A.: Implications for Mid-Atlantic Bight Evolution since the Pliocene. *Mar Geol* **428**, 106287 (2020).
9. Wei, E. & Miselis, J. Shoreface sediment availability offshore of a rapidly migrating, mixed-energy barrier island. in *The Proceedings of the Coastal Sediments 2023* (eds. Wang, P., Royer, E. & Rosati, J. D.) 2903–2916 (World Scientific Publishing Co. Pte. Ltd., 2023).
10. Wehmiller, J. F. *et al.* Molluscan aminostratigraphy of the US Mid-Atlantic Quaternary coastal system: Implications for onshore-offshore correlation, paleochannel and barrier island evolution, and local late Quaternary sea-level history. *Quat. Geochronol.* **66**, 101177 (2021).
11. Thieler, E.R., Himmelstoss, E.A., Zichichi, J.L., and Ergul, A. Digital Shoreline Analysis System (DSAS) version 4.0—An ArcGIS extension for calculating shoreline change: U.S. Geological Survey Open-File Report 2008-1278, http://woodshole.er.usgs.gov/project-pages/DSAS/version4/ (2008).
12. Hapke, C. J., Himmelstoss, E. A., Kratzmann, M. G., List, J. H. & Thieler, E. R. National Assessment of Shoreline Change: Historical Shoreline Change along the New England and Mid-Atlantic Coasts: USGS Open-File Report 2010-1118. https://pubs-usgs-gov.libezp.lib.lsu.edu/of/2010/1118/ (2011).
13. Mariotti, G. & Hein, C. J. Lag in response of coastal barrier-island retreat to sea-level rise. *Nature Geoscience* **15**, 633–638 (2022).
14. Deaton, C. D., Hein, C. J. & Kirwan, M. L. Barrier island migration dominates ecogeomorphic feedbacks and drives salt marsh loss along the Virginia Atlantic Coast, USA. *Geology* **45**, 123–126 (2017).
15. Robbins, M. G., Shawler, J. L. & Hein, C. J. Contribution of longshore sand exchanges to mesoscale barrier-island behavior: Insights from the Virginia Barrier Islands, U.S. East Coast. *Geomorphology* **403**, 108163 (2022).

16. Smith, A. J. *et al.* Compensatory Mechanisms Absorb Regional Carbon Losses Within a Rapidly Shifting Coastal Mosaic. *Ecosystems* 1–15 (2023) doi:10.1007/s10021-023-00877-7.
17. Hutchings, J. A. *et al.* Carbon deposition and burial in estuarine sediments of the contiguous United States. *Global Biogeochem Cy* **34**, (2020).
18. Nichols, M. M. Sediment accumulation rates and relative sea-level rise in lagoons. *Mar Geol* **88**, 201–219 (1989).
19. Finkelstein, K. Backbarrier contributions to a littoral sand budget, Virginia Eastern Shore, USA. *Journal of Coastal Research* **2**, 33–42 (1986).
20. Finkelstein, K. & Ferland, M. A. Back-barrier response to sea-level rise, Eastern Shore of Virginia. *The Society of Economic Paleontologists and Mineralogists* (1987).
21. Shawler, J. L., Ciarletta, D. J., Lorenzo-Trueba, J. & Hein, C. J. Drowned foredune ridges as evidence of pre-historical barrier-island state changes between migration and progradation. *Coastal Sediments Proceedings* 158–171 (2019).
22. Raff, J. L. *et al.* Insights into barrier-island stability derived from transgressive/regressive state changes of Parramore Island, Virginia. *Mar Geol* **403**, 1–19 (2018).
23. Halsey, S. D. Late Quaternary geologic history and morphologic development of the barrier island system along the Delmarva Peninsula of the Mid-Atlantic Bight. (University of Delaware, 1978).
